# A new instrumented method for the evaluation of gait initiation and step climbing based on inertial sensors: a pilot application in Parkinson’s disease

**DOI:** 10.1186/s12984-015-0038-0

**Published:** 2015-05-05

**Authors:** Gianluca Bonora, Ilaria Carpinella, Davide Cattaneo, Lorenzo Chiari, Maurizio Ferrarin

**Affiliations:** Biomedical Technology Department, Found. Don C. Gnocchi Onlus, IRCCS, Via Capecelatro 66, 20148 Milan, Italy; LaRiCe: Gait and Balance Disorders Laboratory, Department of Neurorehabilitation, Found. Don C. Gnocchi Onlus, IRCCS, Via Capecelatro 66, 20148, Milan, Italy; Department of Electrical, Electronic, and Information Engineering - Guglielmo Marconi (DEI), University of Bologna, Viale Risorgimento 2, 40136 Bologna, Italy

**Keywords:** Parkinson’s disease, Gait initiation, Step climbing, Wearable sensors, Anticipatory Postural Adjustments (APAs)

## Abstract

**Background:**

Step climbing is a demanding task required for personal autonomy in daily living. Anticipatory Postural Adjustments (APAs) preceding gait initiation have been widely investigated revealing to be hypometric in Parkinson’s disease (PD) with consequences in movement initiation. However, only few studies focused on APAs prior to step climbing. In this work, a novel method based on wearable inertial sensors for the analysis of APAs preceding gait initiation and step climbing was developed to further understand dynamic balance control. Validity and sensitivity of the method have been evaluated.

**Methods:**

Eleven PD and 20 healthy subjects were asked to perform two transitional tasks from quiet standing to level walking, and to step climbing respectively. All the participants wore two inertial sensors, placed on the trunk (L2-L4) and laterally on the shank. In addition, a validation group composed of healthy subjects and 5 PD patients performed the tasks on two force platforms. Correlation between parameters from wearable sensors and force platforms was evaluated. Temporal parameters and trunk acceleration from PD and healthy subjects were analyzed.

**Results:**

Significant correlation was found for the validation group between temporal parameters extracted from wearable sensors and force platforms and between medio-lateral component of trunk acceleration and correspondent COP displacement. These results support the validity of the method for evaluating APAs prior to both gait initiation and step climbing. Comparison between PD subjects and a subgroup of healthy controls confirms a reduction in PD of the medio-lateral acceleration of the trunk during the imbalance phase in the gait initiation task and shows similar trends during the imbalance and unloading phase of the step climbing task. Interestingly, PD subjects presented difficulties in adapting the medio-lateral amplitude of the imbalance phase to the specific task needs.

**Conclusions:**

Validity of the method was confirmed by the significant correlation between parameters extracted from wearable sensors and force platforms. Sensitivity was proved by the capability to discriminate PD subjects from healthy controls. Our findings support the applicability of the method to subjects of different age. This method could be a possible valid instrument for a better understanding of feed-forward anticipatory strategies.

## Background

The ability to move safely during level walking and stair negotiation is a relevant aspect to guarantee success in performing many activities of daily living (ADLs), such as maneuver over a curb or access to public environments and public transport [1].

Stair negotiation (i.e. ascending and descending stairs) is a demanding and hazardous task for frail people, in particular for older adults and subjects affected by neuromotor disorders, such as Parkinson’s disease (PD). Compared to level walking, stair climbing necessitates of greater range of motion [2-5] and moments at the ankle, knee and hip joints [2,3,6,7], and these requirements can force older adults to use almost their maximal motor capabilities [8] with a consequent increase of the risk of falling. It is reported that falling on stairs is the second more common type of falls in the elderly, and that approximately 75% of all injurious falls on stairs occurs in people aged 65 years or older [9]. Moreover, it was demonstrated that subjects affected by PD have an increased risk of falling compared to healthy controls [10], and that Fear Of Falling (FOF) in the PD population is strongly dependent on walking difficulties, turning hesitation and limited ability to climb stairs [11]. Previous studies showed that these functional limitations are highly associated to alterations in dynamic balance control and to poorly coordinated anticipatory postural adjustments (APAs) prior to voluntary limb movements [12].

APAs represent the transient phase between quiet standing and a dynamic condition chosen voluntarily such as walking, stepping up or down a stair, and over an obstacle [13]. They involve complex interactions between neural and biomechanical factors that serve to maintain postural stability by compensating for destabilizing forces associated with moving a limb [12]. In the case of gait initiation, APAs act to accelerate the center of body mass (COM) forward and laterally over the stance foot by moving the center of pressure (COP) posteriorly and toward the stepping leg. Considering COP displacements, APAs can be divided into two different phases [14]: firstly, the Imbalance Phase characterized by initial displacement of the COP backward and toward the stepping (leading) foot, and then the Unloading Phase in which the COP shifts laterally toward the stance (trailing) foot.

It was demonstrated that APAs are essential to create appropriate initial dynamic conditions [15], that they are affected by modifications of motor behavior due to aging [15,16] and neurological disorders such as Huntington’s chorea [17] and Parkinson’s disease [14,16,18-21], and that they are dependent on the specific task, i.e. stepping forward or upward [13,22-24]. Given the great importance of APAs in the control of dynamic balance, previous studies have suggested to include their analysis to evaluate disease progression in patients with neurological disorders [17], as well as to detect their early clinical signs [18,19].

APAs related to gait initiation are usually recorded using force plates, electromyography, and motion-analysis systems [14,18]. Although all these systems have been proven effective, their cost and complexity limit their application to clinical practice.

Instrumented methods based on low-cost and easy-to-manage inertial sensors were developed in recent years to investigate human balance and postural sway during quiet stance [25,26] and to perform instrumented tests for the evaluation of balance deficits and risk of falling [27,28]. Concerning APAs, inertial solutions were previously developed only for level walking [19,29,30], but not for stair negotiation. Furthermore, in the majority of these studies the analysis was focused only on the imbalance phase, not investigating the subsequent unloading phase that is indeed essential for a correct transition from bi- to mono-pedal stance.

On the basis of the above considerations, in the present study, an easy-to-administer instrumented method based on wearable inertial sensors was developed and applied to healthy subjects and persons affected by PD to analyze the initiation of level walking and step climbing in a typical physical rehabilitation setting: in particular, considering the importance of the unloading process in balance control during the transition from quasi-static to dynamic conditions, a novel algorithm was developed to recognize the initial and final frames of the unloading phase, allowing its subsequent analysis. Aims of this work were to test the validity and sensitivity of the proposed method by: i) validating it against force plate recordings, and, ii) evaluating its ability to differentiate APAs of PD subjects from APAs of healthy controls.

## Methods

### Participants

Twenty healthy subjects (age, mean ± SD: 49.6 ± 17.9 yo, range 23 – 77 years, 10 females) and eleven patients affected by PD (age 72.5 ± 6.8 yo, range 62 – 83 years, 4 females) voluntarily participated in the study.

Healthy subjects were excluded if they presented any neurological disorders, if they used orthotic devices or had artificial joints, or if they were under medication that could affect balance or locomotor functions.

PD subjects were recruited within a group of patients involved in a neuromotor rehabilitation program administered at our rehabilitation institute. They were included in the study if they fulfilled the following inclusion criteria: diagnosis of idiopathic Parkinson’s disease, Hoehn and Yahr (H&Y) stage [31] between 2 and 4, Mini Mental State Examination (MMSE) score [32] higher than 24, ability to stand unsupported more than 10 s, ability to walk for at least 3 m without any walking aid, ability to step up onto a 18-cm high step. Patients were clinically rated by a trained examiner on the H&Y scale and on the Motor Section III of the Unified Parkinson’s Disease Rating Scale (UPDRS) [33] immediately before the beginning of the experimental sessions. Demographic and clinical characteristics of PD subjects are reported in Table 1. Patients were tested while they were on their routine therapy.

**Table 1:** **Subjects’ characteristics at the time of the study**

**Subject**	**Gender**	**Age**	**Disease duration**	**H&Y**	**UPDRS III**
	**(M/F)**	**(years)**	**(years)**	**stage**	**score**
P1	M	71	17	2	27
P2	F	83	7	2.5	15
P3	F	62	8	2	9
P4	M	79	9	3	23
P5	M	72	5	3	22
P6	M	65	6	2	9
P7	M	72	7	2.5	12
P8	M	66	12	2.5	18
P9	F	72	5	2.5	22
P10	F	82	7	2.5	20
P11	M	74	6	2	17
Mean	7 M / 4 F	72.5	8.1	2.4	17.6
SD		6.8	3.6	0.4	5.9

*H&Y: 1÷5; 5 maximum disability. UPDRS III: 0÷56; 56 maximum motor impairment.*

All the 20 healthy subjects and a subgroup of 5 PD patients (age 73.4 ± 6.1 yo, range 65 – 82 years, 2 females) got involved in a validation group (VG) for investigating the validity of the proposed method.

The eleven oldest subjects of the twenty healthy volunteers (age 66.6 ± 6.1 yo, range 60 – 77 years, 5 females) were selected as healthy controls (HC) for the comparative analyses. The ages of HC were comparable to those of PD subjects (p-value = 0.09).

All the participants signed informed consent forms approved by the local Ethical Committee.

### Experimental equipment

All PD subjects and healthy controls wore 2 inertial sensors (TMA, Tecnobody, Dalmine, Italy) embedding a 3D accelerometer (range ± 5 g), and a 3D gyroscope (range ± 2000 °/s). Linear acceleration and angular velocity data were sampled at 50 Hz and transmitted to a remote PC through a Bluetooth wireless connection for subsequent offline analysis.

**Figure 1:**
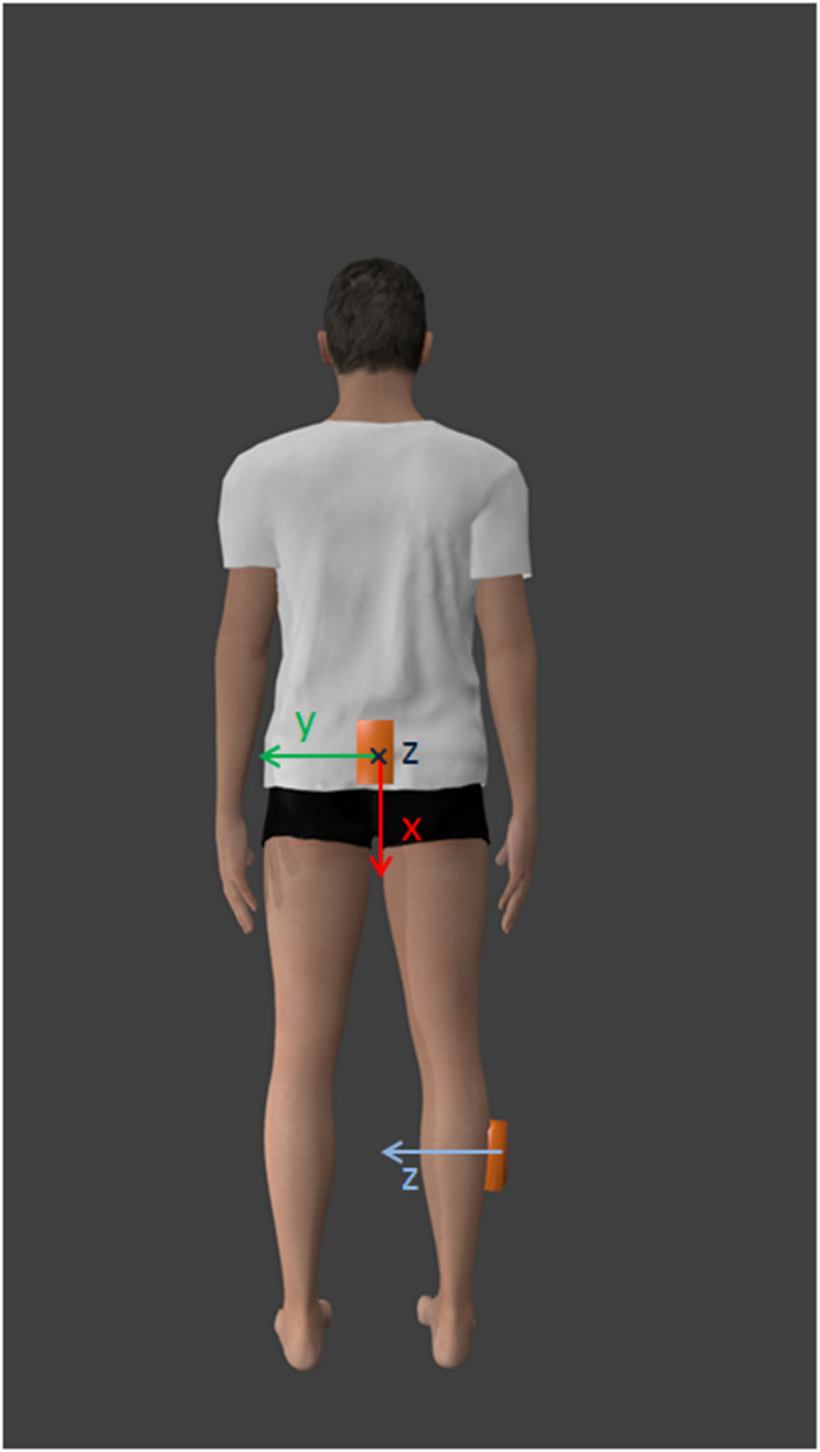
Wearable inertial sensors placement.

As shown in Figure 1, one sensor was placed on the posterior trunk, in correspondence to L2-L4 vertebra, with the sensing axes (x, y and z) oriented along the body vertical, medio-lateral (ML) and antero-posterior (AP) directions, respectively. The second sensor was placed proximally on the lateral aspect of the shank of the first stepping leg with the z-axis oriented along the limb medio-lateral direction. Sensors were fixed over clothing through anti-slip elastic bands.

**Figure 2:**
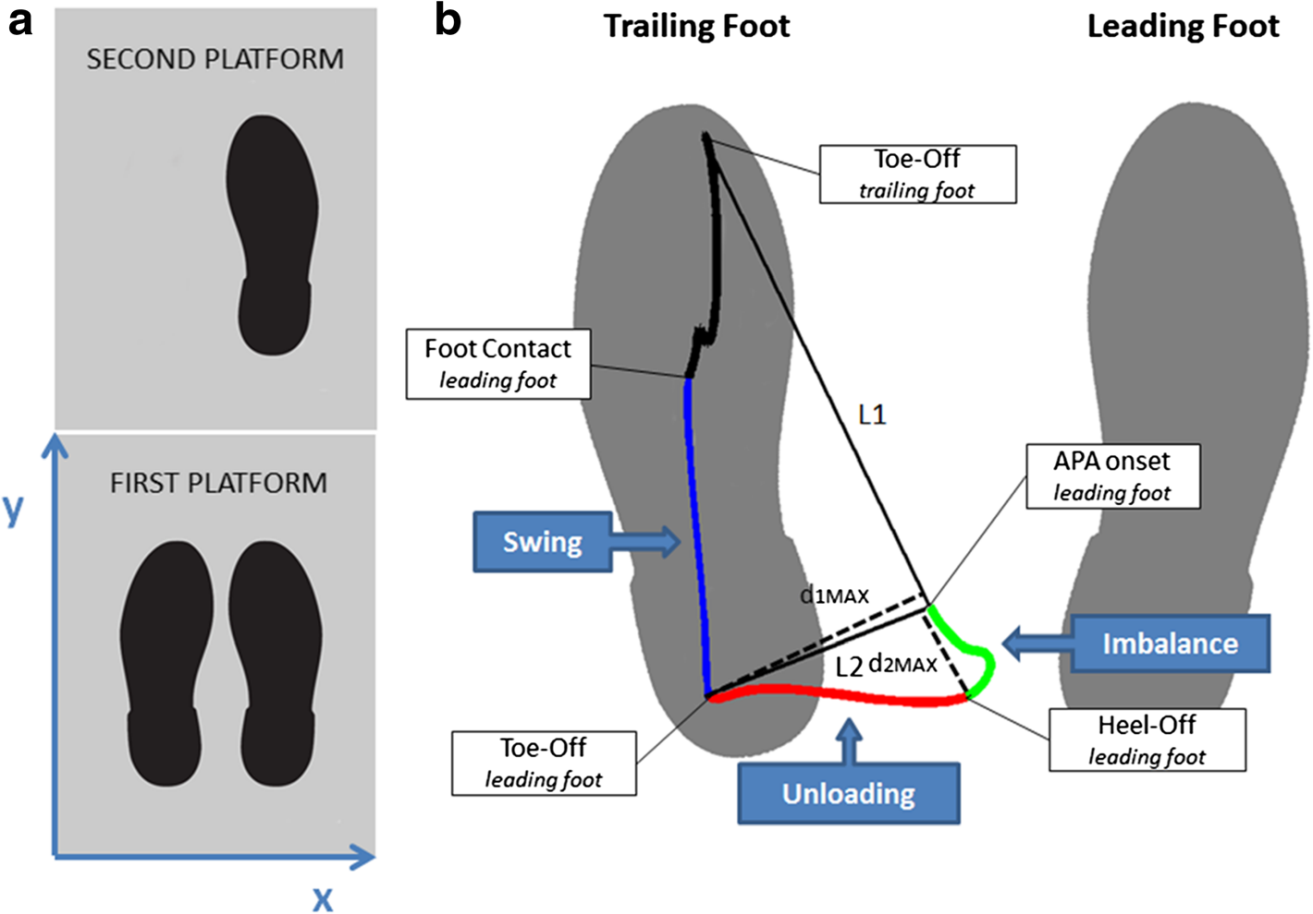
Laboratory setup for the analysis of COP displacement during APAs. **a)** Placement of the two force plates **b)** COP displacement in the medio-lateral (x) and antero-posterior (y) directions during the gait initiation process in a healthy subject. APA onset, Heel-Off, Toe-Off, and Foot Contact instants of the leading foot and the Toe-Off instant of the trailing one are reported. Imbalance (from APA onset to heel-off of the leading foot), unloading (from heel-off to toe-off of the leading foot), and swing (from toe-off to foot contact of the leading foot) phases are indicated. L1 line passing through the points representing the COP position at APA onset and at the toe-off of the trailing foot instants, and L2 line passing through the points representing the COP position at APA onset and at the toe-off of the leading foot instants are drawn. d_1MAX_ represents the maximal distance from L1 attained by the COP (corresponding to toe-off of the leading foot), while d_2MAX_ represents the maximal distance from L2 attained by the COP (corresponding to heel-off of the leading foot).

Ground reaction forces and COP displacement of VG subjects tested in the motion analysis laboratory were measured by means of two force plates (Kistler Gmbh, Winterthur, Switzerland) with a sampling frequency of 800 Hz, considered as gold standard for APAs analysis (Figure 2a).

### Experimental protocol

Subjects were asked to perform two different transitional tasks: 1) quiet standing to level walking (gait initiation); and 2) quiet standing to single step climbing (step climbing). Three consecutive repetitions for each task were recorded in the above mentioned order.

At the beginning of each trial, subjects stood upright for 10 s in a comfortable position with the arms laying on their sides, wearing flat shoes with no heels: no given distances between the feet were imposed, in accordance with protocols developed in previous studies about anticipatory postural strategies preceding stepping upward [22-24] and over an obstacle [13]. As soon as they received a vocal command from the experimenter, participants started the task execution. In the first task, subjects had to walk along a straight trajectory for about 3 m, while in the second one, they were asked to step up onto the first level of a two-step staircase. Each step measured 18 cm in height, 38 cm in width, and 34 cm in depth. The step dimensions were chosen to be among the most frequently encountered in public places and new residential buildings.

Both gait initiation and step climbing were executed by all the participants, both healthy and PD subjects, starting with their right leg, as reported to be the dominant one, at self-selected speed.

Six of the eleven PD patients were tested in a typical rehabilitation setting before the beginning of their conventional physiotherapy session while all the members of the validating group, composed of the 20 healthy subjects and the five PD patients who accepted to be tested outside the rehabilitation gym, executed the tasks in a motion analysis laboratory equipped with two force plates embedded in the floor (see previous section). In the laboratory, VG subjects were required to start the gait initiation task with both feet on the first force plate and then to step forward on the second platform, while in the step climbing task, they were asked to stand upright with both feet on the first force plate and then stepping up onto the lower step of the staircase placed in front of them on the second force plate.

### Data processing

After data recording, signals from force plates and inertial sensors were processed to analyze the anticipatory postural adjustments preceding gait initiation and step climbing.

COP displacements recorded from the force plate were filtered with a fourth order, zero-lag, low-pass Butterworth filter with a cut-off frequency of 10 Hz [19]. COP trajectory and vertical ground reaction force were then used to subdivide each task into the initial quasi-static APA phase, made up of the imbalance and unloading phases, and the subsequent dynamic phase corresponding to the swing of the first leading foot. For this purpose, 4 instants were automatically identified by a dedicated algorithm and visually checked through an interactive software: 1) APA onset, 2) heel-off, 3) toe-off, and 4) foot contact of the leading foot (see Figure 2b). In particular, APA onset was detected with a threshold-based algorithm applied to the COP medio-lateral displacement with the threshold set as twice the standard deviation (SD) of the signal during the quiet standing period preceding task initiation, as proposed in [19]. Heel-off and toe-off of the leading foot were detected as proposed in [14]: referring to Figure 2b, the toe-off of the trailing limb was detected as the last frame of the first force platform signal; then the toe-off of the leading foot was recognized as the instant in which the position of the COP attained the maximal distance (d_1MAX_) from the line passing through the two points representing the APA onset and the toe-off of the trailing limb (L1). Finally, the heel-off of the leading foot was computed as the frame in which the COP position attained the maximal distance (d_2MAX_) from the line passing through the two points representing the APA onset and the toe-off of the same foot (L2). The foot contact of the leading limb was recognized as the instant when the vertical ground reaction force of the second platform exceeded a threshold of 6.5% of body weight, as suggested in [34]. The same detection method was adopted for both the gait initiation and the step climbing tasks.

**Figure 3:**
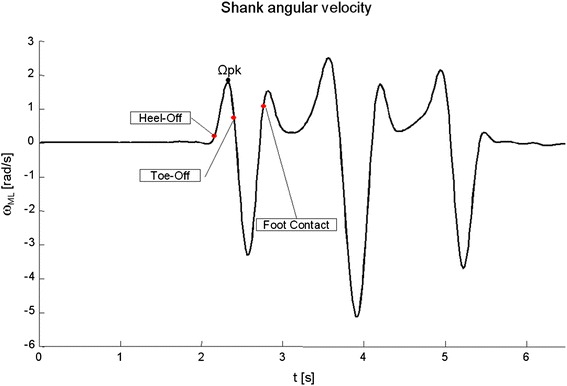
Angular velocity of the shank respect to its medio-lateral axis: the first peak of the signal (Ωpk) is reported while red dots correspond to heel-off, toe-off and foot contact of the leading limb as recognized by the proposed detection algorithm.

Temporal instants were then extracted from the wearable inertial system data. The acceleration signals recorded at trunk level were transformed to horizontal-vertical coordinate system [35] and filtered using a fourth order, zero-phase, low-pass Butterworth filter with a cut-off frequency of 3.5 Hz, as proposed by Mancini et al. [19]. The same filter was also applied to angular velocity data recorded by the sensor placed on the shank. The APA onset was detected with a threshold-based algorithm applied to the ML acceleration of the trunk sensor [19] with the threshold set as the SD of the signal during the quiet standing period preceding task initiation, multiplied by a factor A. The shank angular velocity around the ML axis was used to identify heel-off and toe-off instants, as shown in Figure 3. In particular, the first peak of the signal (Ωpk) was detected, then the heel-off was estimated as the first instant, following the APA onset, at which the angular velocity became higher than Ωpk value multiplied by a factor H. Toe-off was identified as the first instant, following the peak, at which the signal became lower than Ωpk multiplied by a factor T. The initial calibration of the thresholds was performed considering the data collected on VG subjects, tested in the motion lab with both force plate and inertial sensors. During the calibration procedure, different sets of temporal instants were computed by varying the multiplicative parameters A, H and T. In particular, factor A was varied between values 1 and 5 with unitary incremental steps, while H and T were varied between 0 and 1 with incremental steps equal to 0.01 and 0.05 respectively. For each set of instants and for each subject, the mean absolute errors (MAEs) between instants calculated from force plates data and frames extracted from inertial sensors signals were computed and averaged among all subjects. The final values of A, H and T were then chosen as those which minimized the averaged errors. Finally, the foot contact instant was estimated as the median point between the second peak of the angular velocity and the preceding zero-crossing event; the point was chosen as the one that minimize MAEs. The set of extracted thresholds was then applied to all subjects, including the PD patients tested in the rehabilitation gym, without any further usage of the force plate.

After the events detection algorithm was applied, the following spatio-temporal parameters were computed from both COP displacement and trunk accelerations:

#### Temporal parameters

- Imbalance phase duration: from APA onset to the heel-off of the leading foot.
- Unloading phase duration: from the heel-off to the toe-off of the leading foot.
- APA duration: from APA onset to the toe-off of the leading foot, as the sum of imbalance phase and unloading phase durations.
- Swing phase duration: from the toe-off to the foot contact of the leading foot.
- Step duration: from APA onset to the foot contact of the leading foot.

#### Spatial parameters

- Imbalance phase amplitude in ML (AP) direction: i) calculated from force plate data as the difference between COP ML (AP) position at heel-off and COP ML (AP) position at APA onset; ii) estimated from inertial sensors signals as the difference between trunk ML (AP) acceleration measured at heel-off and trunk ML (AP) acceleration measured at APA onset.
- Unloading phase amplitude in ML (AP) direction: i) calculated, from force plate data, as the difference between COP ML (AP) position at toe-off and COP ML (AP) position at heel-off; ii) estimated, from inertial sensors signals, as the difference between trunk ML (AP) acceleration measured at toe-off and trunk ML (AP) acceleration measured at heel-off.

Spatial parameters were computed only for the APA phase, due to the quasi-stationary condition required by Moe-Nilssen [35]. Recognizing that, during APAs, COM and COP typically act as they were reciprocally linked (i.e. in the imbalance phase, COM moves forward and laterally over the stance foot, while COP moves posteriorly toward the stepping foot) and considering the results already reported in literature [19], we hypostasized that i) lower trunk accelerations are significantly correlated with COP displacements during APAs and that ii) lower trunk acceleration data can therefore be used to estimate force platform variables.

### Statistical analysis

For each subject, variables were averaged over the three trials of each test. Parametric statistical tests were used for the analysis, as data normality and homoscedasticity were confirmed by Shapiro-Wilk’s W test and Bartlett’s test, respectively.

Mean absolute errors (MAEs) between temporal instants extracted from force plate data and inertial sensors were compared among young adults, older healthy subjects, and PD patients by using ANOVA test.

The concurrent validity of the proposed method for evaluating APAs was investigated through a linear regression analysis between the parameters extracted from the force plate and the correspondent ones computed from inertial sensors, as proposed in previous studies [19,25] . Pearson’s correlation coefficient r and the related p-value were therefore calculated considering data recorded from the VG subjects tested in the motion lab.

For each parameter, a Student’s *t*-test was adopted to detect differences between PD patients and the subset of comparable aged control subjects (HC). Finally, comparisons of the above mentioned temporal and spatial parameters were performed between the two tasks (gait initiation and step climbing) by using paired *t*-test.

The level of significance was set at 0.05 for all the conducted analyses.

All the analyses were performed with R (R Foundation for Statistical Computing, Vienna, Austria).

## Results

### Validity of the method

Validity of the proposed method was assessed considering data related to the VG subjects tested with both inertial sensors and force plates.

Table 2 shows the values of the multiplicative factors (A, H, and T) used by the threshold-based algorithm for the event detection procedure, the correspondent mean absolute errors (MAEs) between instants computed from inertial sensor signals and frames identified from force plate data, and the percentage errors referred to the step duration. It is possible to notice that the highest error (6.3%) is associated with the detection of the APA onset in the step climbing task: no statistically significant differences in MAEs were noticed between the two tasks (p = 0.79) and between younger adults (<60 yo), elderly subjects (>60 yo), and PD patients (p = 0.73).

**Table 2:** **Mean absolute error of event detection between inertial sensors and force plates**

	**Gait initiation**		**Step climbing**	
APA onset	A = 2	0.05 ± 0.03 (5.0%)	A = 2	0.09 ± 0.05 (6.3%)
Hell-Off	H = 0.07	0.07 ± 0.03 (5.8%)	H = 0.08	0.08 ± 0.05 (5.6%)
Toe-Off	T = 0.25	0.05 ± 0.03 (4.1%)	T = 1	0.06 ± 0.03 (4.2%)
Foot Contact	-	0.06 ± 0.08 (5.0%)	-	0.07 ± 0.04 (4.9%)

Mean absolute error of event detection (mean ± SD [s]) and, in brackets, percentage error referred to the step duration (from APA onset to foot contact).

*Multiplicative coefficients (A, H and T) set in the threshold-based algorithm for the event detection are reported.*

As reported in Table 3, a significant linear correlation was found between COP medio-lateral displacements and the correspondent trunk accelerations in both tasks, while no correlation was found for the antero-posterior features. A significant linear correlation between the two methods was also noticed considering the duration of the whole test and its phases.

**Table 3:** **Linear correlation between inertial sensors and force plates measures**

	**Gait initiation**			**Step climbing**		
	**AP**	**ML**	**Δt**	**AP**	**ML**	**Δt**
Imbalance	0.20	0.81*	0.78*	0.36	0.81*	0.77*
Unloading	0.15	0.65*	0.48*	0.19	0.81*	0.62*
APA	0.16	0.69*	0.70*	0.14	0.65*	0.83*
Swing	-	-	0.73*	-	-	0.77*
Step	-	-	0.82*	-	-	0.83*

Pearson’s correlation coefficients (r) between phase durations (Δt) measured by force platform and wearable sensors and between COP displacement and trunk acceleration in the antero-posterior (AP) and medio-lateral (ML) directions.

Significant correlations (p-value < 0.05) are shown with*.

### Differences between PD subjects and comparable aged controls (sensitivity)

To assess the sensitivity of the proposed method, comparison between PD patients and healthy controls (HC) was performed considering only temporal and medio-lateral spatial parameters extracted from the inertial sensors, as we proved their validity in the former analysis.

**Figure 4:**
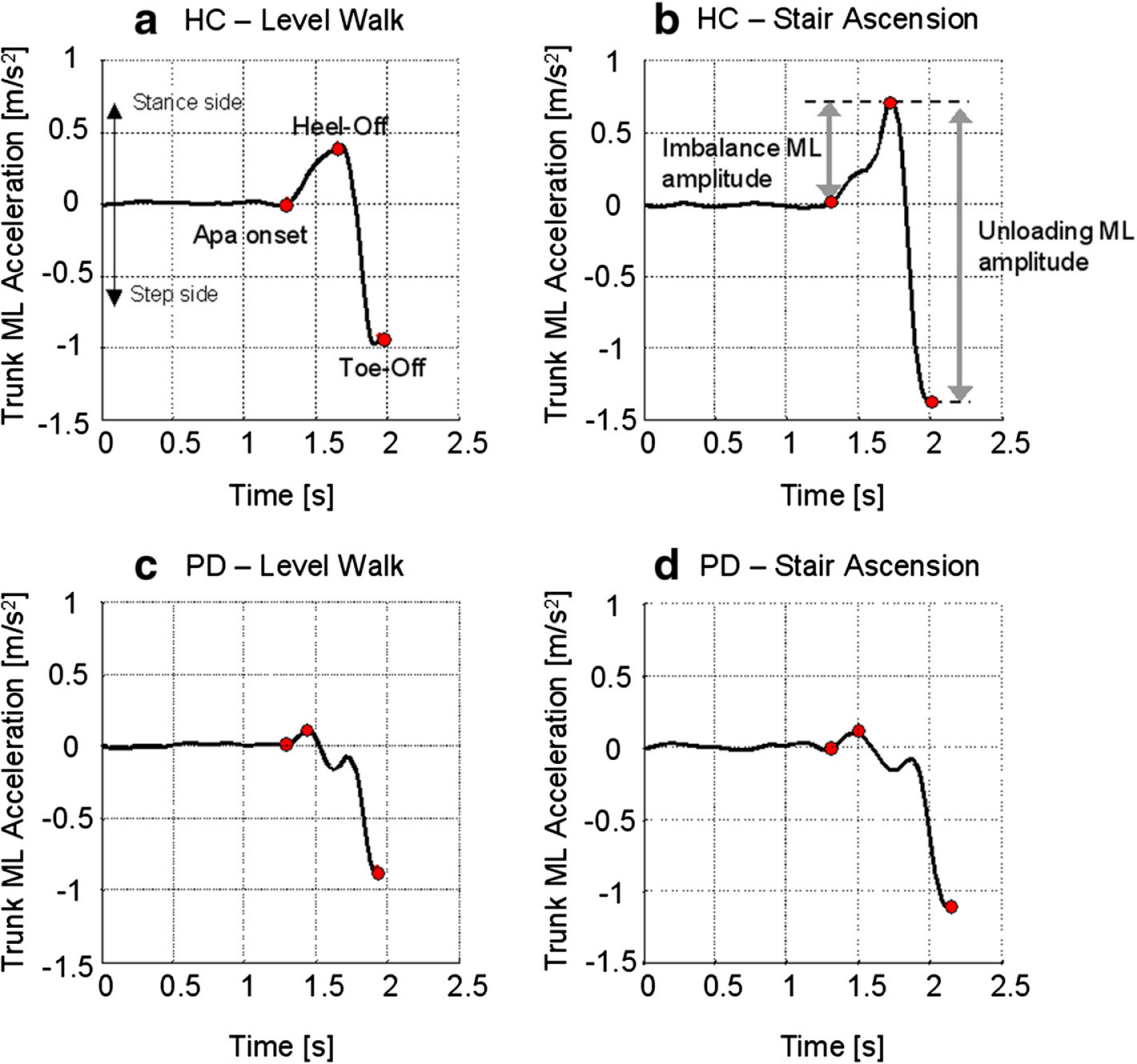
Trunk acceleration during APAs in two representative subjects: control subject **(a, b)** and PD subject **(c, d)**. Signals collected during the initiation of level walk **(a, c)** and stair ascension **(b, d)** are shown. APA onset, heel-off and toe-off are reported as red marks.

Collected results are shown in Table 4, while Figure 4 shows examples of the trunk acceleration signal recorded from a representative control and a PD subject in the level walk and step climbing tasks. Regarding the imbalance phase, trunk ML acceleration was significantly smaller in PD subjects with respect to HC both in level walking and step climbing. Furthermore, as shown in Figure 4a-b, control subjects showed a significant increase of the medio-lateral acceleration during step climbing with respect to level walking (Level walking: 0.19 ± 0.08 m/s^2^; Stair climbing: 0.26 ± 0.13 m/s^2^; p = 0.01). No such a difference was found in the PD group (Level walking: 0.08 ± 0.12 m/s^2^; Step climbing: 0.09 ± 0.15 m/s^2^; p = 0.78) (see Figure 4c-d). Regarding the unloading phase, a significant reduction of the ML acceleration in PD subjects was found in the step climbing task but not in the level walking.

**Table 4:** **Comparison of parameters extracted from inertial sensors in PD patients and healthy controls (HC)**

		**Gait initiation**			**Step climbing**		
		**HC**	**PD**	**p-value**	**HC**	**PD**	**p-value**
Imbalance	ML	0.19 ± 0.08	0.08 ± 0.12	0.02*	0.26 ± 0.13	0.09 ± 0.15	< 0.01*
	Δt	0.40 ± 0.17	0.30 ± 0.20	0.70	0.47 ± 0.18	0.38 ± 0.14	0.20
Unloading	ML	−0.65 ± 0.37	- 0.42 ± 0.32	0.14	−0.76 ± 0.49	- 0.56 ± 0.42	0.03*
	Δt	0.30 ± 0.06	0.37 ± 0.15	0.17	0.30 ± 0.05	0.32 ± 0.17	0.56
APA	ML	−0.53 ± 0.24	- 0.57 ± 0.37	0.45	−0.57 ± 0.37	- 0.52 ± 0.46	0.41
	Δt	0.70 ± 0.15	0.85 ± 0.66	0.63	0.77 ± 0.20	0.84 ± 0.57	0.31
Swing	Δt	0.49 ± 0.07	0.42 ± 0.07	0.03*	0.65 ± 0.10	0.62 ± 0.13	0.30
Step	Δt	1.23 ± 0.11	1.28 ± 0.37	0.70	1.33 ± 0.10	1.36 ± 0.25	0.75

Trunk acceleration in the medio-lateral direction (ML; mean ± SD [m/s^2^]) and durations (Δt; mean ± SD [s]) of the different phases and the complete test in the two different transitional tasks.

Significant differences (p-value < 0.05) are marked with*.

As for temporal parameters, a statistically significant difference between the two groups was found only in swing phase duration that was lower in PD subjects with respect to HC. In addition, the correlations between the investigated parameters and the UPDRS III scores resulted to be not significant; this result is in accordance with [19].

All these findings are enforced by data extracted through the force plates from the HC subjects and the 5 PD patients enrolled in the validation group (Table 5). The only exception was related to the swing phase duration which was slightly smaller in PD subjects, but not statistically different from HC as it was instead shown by acceleration data. This was probably due to the small sample size. When comparing step-climbing with level walking, similarly to the results obtained from wearable inertial sensors, an increase of the medio-lateral COP displacement was observed in healthy subjects (Level walking: 2.17 ± 0.70 cm; Stair climbing: 2.48 ± 0.97 cm; p = 0.04), but not in the five patients (Level walking: 1.35 ± 0.74 cm; Stair climbing: 1.60 ± 0.59 cm; p = 0.27).

**Table 5:** **Comparison of parameters extracted from force plates in the validation group (VG)**

		**Gait initiation**			**Step climbing**		
		**HC**	**PD**_**VG**_	**p-value**	**HC**	**PD**_**VG**_	**p-value**
Imbalance	ML	2.17 ± 0.70	1.35 ± 0.74	0,04*	2.48 ± 0.97	1.60 ± 0.59	0.02*
	Δt	0.30 ± 0.03	0.30 ± 0.09	0.77	0.49 ± 0.16	0.40 ± 0.13	0.29
Unloading	ML	−9.35 ± 1.85	−7.41 ± 1.50	0.06	- 9.55 ± 1.94	- 7.86 ± 0.62	0.02*
	Δt	0.38 ± 0.09	0.40 ± 0.19	0.82	0.37 ± 0.07	0.53 ± 0.26	0.25
APA	ML	−7.18 ± 1.40	−6.05 ± 1.36	0.16	- 6.95 ± 1.32	- 6.26 ± 0.31	0.13
	Δt	0.68 ± 0.09	0.69 ± 0.28	0.95	0.86 ± 0.17	0.93 ± 0.17	0.7
Swing	Δt	0.48 ± 0.07	0.44 ± 0.04	0.24	0.64 ± 0.08	0.62 ± 0.04	0.43
Step	Δt	1.19 ± 0.14	1.18 ± 0.29	0.96	1.43 ± 0.18	1.42 ± 0.12	0.86

Comparison between the healthy controls (HC) and the subgroup of 5 PD patients enrolled in the validation group (PD_VG_).

*COP displacement in the medio-lateral direction (ML; mean ± SD [cm]) and durations (Δt; mean ± SD [s]) of the different phases and the complete test in the two different transitional tasks. Data of the healthy controls (HC) and the subgroup of 5 PD patients enrolled in the validation group (PD*_*VG*_ ) are reported. Significant differences (p-value < 0.05) are marked with*.

## Discussion

In the present study an instrumented method based on wearable inertial sensors was developed and applied on healthy subjects and on persons affected by PD to analyze the initiation of gait and step climbing. To our knowledge this is the first study aimed at comparing the APAs prior to level walking and step climbing through wearable inertial sensors, and it represents the first attempt to investigate differences between the two tasks in a group of PD subjects under their usual medication state. Specific aims of this work were: i) validating it against force plate recordings, and, ii) evaluating its ability to differentiate APAs of PD subjects from APAs of healthy controls. These two different aspects will be discussed separately.

### Methodological aspects and validity of the procedure

The first objective of this work was to develop a method that offers the possibility to study APAs prior to level walking and stair climbing directly in a typical physical rehabilitation setting, without the necessity of expensive equipment such as force platforms. For this reason it was chosen to use low-cost, easy-to-use wearable inertial sensors, as previously proposed by other authors for the investigation of the gait initiation process [19,30]; in these studies a single inertial measurement unit was used and the analysis was limited to lower trunk acceleration during imbalance phase, in which COP shifts backward an toward the stepping foot. To our knowledge, no studies exist about the use of inertial sensors to analyze the subsequent unloading phase (from the heel-off to the toe-off instant of the leading leg) which implies COP shift toward the trailing foot. Considering that correct unloading is essential for the maintenance of dynamic balance during the transition from bi- to mono-pedal stance, in the present work we decided to include this specific aspect into the analysis. For this reason, a second sensor was applied on the lower limb to allow an easier detection of heel-off and toe-off frames from shank angular velocity. The lack of easy-detectable changes in both acceleration and angular velocity signals in correspondence of heel-off and toe-off events compelled us the implementation of a threshold-based automated algorithm for the recognition of heel-off and toe-off temporal instants. The developed solution required an initial calibration of the thresholds on the basis of force plate data. After this preliminary set-up, the algorithm was applied to all subjects without any further usage of force plates.

The proposed procedure was validated for healthy subjects with different ages (from 23 to 77 years) and a subgroup of 5 PD patients by means of a comparison with force plates data, considered as a gold standard. Analysis of the temporal frames extracted with the two systems (i.e. APA onset, heel-off, toe-off and the subsequent foot contact of the leading foot) revealed mean absolute errors (MAEs) ranging from 0.05 s to 0.09 s. At our knowledge, no previous studies evaluated errors in the estimation through wearable inertial sensors of specific movements of the leading limb; in absence of term of comparisons, we considered the reported MAEs acceptable for the aim of the present study. No statistically significant differences in MAEs were recognized after comparisons between level walking and stair climbing, and between young adults (age < 60 yo), healthy elderly subjects (age ≥ 60 yo), and PD patients. This result suggests that the method is applicable with comparable accuracy to adults with different age and subjects affected by PD in both tasks.

Importantly, linear regression analysis related to both level walking and stair climbing revealed a significant positive correlation between temporal parameters (i.e. duration of the step and of each phase of the test) extracted from inertial sensor and the same variables computed from force plate data. Regarding spatial parameters, the amplitude of APAs measured from COP displacement and estimated from acceleration signals in medio-lateral direction were significantly correlated, in accordance with [19,30]. No such a correlation was found considering the antero-posterior direction. This difference between AP and ML directions could be ascribed to the following consideration. While medio-lateral movements characterizing APAs can be considered mono-segmental (i.e. the entire body moves laterally around the feet to prepare the subsequent step, using mainly the ankle joint), antero-posterior movements can be considered multi-segmental, involving not only the ankle but also the hip joints, especially in elderly subjects [36]. For this reason, the link between COP AP displacement and trunk AP acceleration might result more complex than that observed in the ML direction, thus explaining the lack of correlation found in the present results.

In summary, the present results suggested the validity of the proposed method for evaluating temporal aspects and medio-lateral features of the APAs preceding both gait initiation and step climbing.

### Method’s application on PD subjects

The method was applied on a group of PD subjects and the results were compared to those related to healthy controls (HC) of comparable age. Only temporal parameters and spatial variables related to ML direction were considered, because we formerly proved their validity on the selected validation group. As a consequence of the good correlation with the force platform and of the applied transformation to horizontal-vertical coordinate system [35], the trunk acceleration pattern registered by the waist-worn sensor can be considered reciprocally linked to the COP displacement pattern during APAs, as previously proposed by other authors [19,29,30].

In the case of level walking, a significant reduction of trunk medio-lateral acceleration was observed in PD subjects during the imbalance phase, confirming that APAs related to gait initiation are hypometric in PD [16,19,20]. On the contrary, ML amplitude of the unloading phase was similar in both groups, confirming the results obtained by Mazzone et al. [21] on force plate data.

The feed-forward postural preparation during the imbalance phase has the primary consequence of determining the COM disequilibrium needed for lowering the load of the stepping leg and allowing its forward and upward progression; a reduction of that perturbation could be therefore seen as an attempt to minimize postural instability [18,19,37,38]. Analysis of temporal aspects of gait initiation did not reveal any difference between the two groups both in imbalance and unloading phase. This result is in contrast to that found by Crenna et al. [14] and Halliday et al. [16] who demonstrated a significant prolongation of both phases in PD patients. This discrepancy may be explained by differences in the medication state of the participants, as subjects included in the cited studies were in OFF-medication state while in contrast in the present work PD subjects were tested while they were under their routine therapy.

In addition to the reduction of the ML trunk acceleration, our results revealed a significantly shorter duration of the first step swing phase for the PD group. Even though step length was not considered in the present study, previous works demonstrated a significant reduction of this parameter during gait initiation [16,23,29]. On the basis of this consideration, it can be speculated that the reduction in step duration found in the present study could be related to a shortening of the stride length and to an increase in cadence that are typical of PD patients [39].

Furthermore, a significant reduction in medio-lateral amplitude of the unloading phase was also present, suggesting that APAs prior to step climbing are more compromised with respect to those preceding gait. Previous electromyographic studies demonstrated that the preparation to stepping up is characterized by a greater activity of hip abductor muscle and an earlier onset of gluteus medius [24]; hence, the greater request at the expense of the hip muscles, that is indeed weaker in PD subjects [40], could partly explain the significant reduction in medio-lateral acceleration that was noticed both in the unloading and in the imbalance phase prior to step climbing.

Interestingly, a further difference between PD and HC groups emerged from the comparison between level walking and stair ascending APAs; in particular, in healthy subjects, the medio-lateral amplitude of unloading phase prior to stepping upward was significantly larger with respect to that preceding stepping forward, as found in previous studies [13,24]. This finding could be ascribed to the fact that stepping up is more challenging for ML balance control than level walking, as it presents the additional constraint of not stumbling with the leading foot on the step, and this can be the reason for larger medio-lateral unloading, which ensures that center of mass is safely within the contact area of the supporting foot [13]. No such a difference was found in PD subjects who showed similar medio-lateral amplitude of unloading phase in both tasks. This result is particularly interesting taking into account the already published findings on healthy subjects; in those studies, the ability to scale the anticipatory postural strategies on the basis of task requirements [23,41,42], and the differences in APAs preceding stepping over an obstacle or up a stair [13,22-24] have been well documented. These in turn can be considered as a mechanism adopted by the central nervous system to safeguard balance during different transitional tasks. On the contrary, the absence of scaling found in the PD group could imply a difficulty to adapt feed-forward anticipatory strategies to different stepping task, that seems to be consistent with deficits in neural control, proprioception [43,44] and muscle weakness, mainly of the hip joint [40].

Such reduced adaptability may have a role in step climbing limitations that are typical of PD patients, with a consequent increase of anxiety and risk of falling [11].

Importantly, the above results were confirmed by force plate data recorded from elderly controls (HC) and the subgroup of 5 PD patients tested in the motion lab. Taken altogether, these results confirmed the validity of the proposed method for evaluating APA preceding gait initiation and step climbing.

### Limitation of the study

There are some limitations that need to be addressed regarding the present study. A first limitation is represented by the small number of subjects included in this study; the proposed method should be applied on a greater number of patients in order to confirm these preliminary results. Secondly, the validity of the proposed procedure was performed on healthy subjects and 5 PD patients. In fact only 5 of all the tested PD subjects gave their consent to perform the test outside the rehabilitation gym in the motion laboratory equipped with force plates. Moreover, considering that the aim of the present work was to verify the applicability of the method directly in a physical rehabilitation setting, we considered the described validation procedure suitable for a first pilot study. Anyway, future studies are warranted to validate the method on a greater sample of PD patients and, possibly, on subjects affected by other different neurological disorders such as Multiple Sclerosis, and to test the reliability of the proposed variables. A third limitation of the study is represented by the fact that no given distances between the feet were imposed in both the tasks. Spontaneous feet placement on the floor with no constraints was allowed in accordance with previous studies on the adaptation of anticipatory postural strategies for stepping upward [22-24] and over an obstacle [13], and it was intended to guarantee the maximal level of comfort, self-confidence, and safety prior to attempt the requested complex transitional tasks without walking aid. Finally, a further investigation to define the minimum significant detectable changes is desirable for a future application of the method to evaluate the course of the disease and possible rehabilitation effects.

## Conclusion

In summary, the results of the present study showed that the proposed method based on inertial sensors i) is applicable in clinical settings to evaluate APAs preceding both gait initiation and step climbing, and ii) is able to discriminate APAs of PD subjects under their usual medication state from those of healthy controls of comparable age. In particular, PD subjects showed altered APAs in both gait initiation and step climbing, with the latter task showing more pronounced alterations. Moreover, difficulties in modifying feed-forward anticipatory strategies on the basis of the specific transitional task was demonstrated in PD group. Validity of the method was verified through the comparison with force plate data. Even though caution must be taken due to the small sample size, these preliminary findings suggest that the proposed procedure could be a fast, easy-to-manage and cost effective solution for a quantitative characterization of APAs in PD patients in those clinical settings where force platforms are usually not available.
